# Body Composition of Patients Undergoing Radical Cystectomy for Bladder Cancer: Sarcopenia, Low Psoas Muscle Index, and Myosteatosis Are Independent Risk Factors for Mortality

**DOI:** 10.3390/cancers15061778

**Published:** 2023-03-15

**Authors:** Simon U. Engelmann, Christoph Pickl, Maximilian Haas, Sebastian Kaelble, Valerie Hartmann, Maximilian Firsching, Laura Lehmann, Miodrag Gužvić, Bas W. G. van Rhijn, Johannes Breyer, Maximilian Burger, Roman Mayr

**Affiliations:** 1 Department of Urology, St. Josef Medical Center, University of Regensburg, Landshuterstraße 65, 93053 Regensburg, Germany; 2 Department of Surgical Oncology (Urology), Netherlands Cancer Institute, Antoni van Leeuwenhoek Hospital, 1066 CX Amsterdam, The Netherlands

**Keywords:** sarcopenia, body composition, urothelial carcinoma, bladder cancer, cystectomy, cachexia

## Abstract

**Simple Summary:**

Assessment of body composition in bladder cancer patients has not been sufficiently performed in larger patient cohorts. In other tumor entities, implications of the prognostic value of certain body composition traits have been made. The aim of our retrospective single-center study on 657 patients was to assess different muscle and adipose tissue indices in order to identify those relevant as prognostic factors for overall survival (OS) and cancer-specific survival (CSS) in bladder cancer patients. We also aimed to assess different thresholds described in the literature for other tumor entities. Unification and consensus of definitions are urgently needed in this field of research. We identified sarcopenia, low psoas muscle index (PMI), and myosteatosis as independent risk factors for OS and CSS.

**Abstract:**

Background: We assessed a wide array of body composition parameters to identify those most relevant as prognostic tools for patients undergoing radical cystectomy (RC) due to bladder cancer (BC). Methods: In this retrospective, single-center study, preoperative computed tomography (CT) scans of 657 patients were measured at the level of the 3rd lumbar vertebra (L3) to determine common body composition indices including sarcopenia, myosteatosis, psoas muscle index (PMI), subcutaneous and visceral fat index (SFI and VFI), visceral-to-subcutaneous fat ratio (VSR), and visceral obesity. Predictors of overall survival (OS) and cancer-specific survival (CSS) were identified in univariate and multivariate survival analysis. Results: Sarcopenia and a low PMI were independently associated with shorter OS (Sarcopenia: HR 1.30; 95% CI 1.02–1.66; *p* = 0.04 and a low PMI: HR 1.32; 95% CI 1.02–1.70; *p* = 0.03) and CSS (Sarcopenia: HR 1.64; 95% CI 1.19–2.25; *p* < 0.01 and a low PMI: HR 1.41; 95% CI 1.02–1.96; *p* = 0.04). Myosteatosis, measured as decreasing average Hounsfield units of skeletal muscle, was an independent risk factor for OS (HR 0.98; 95% CI 0.97–1.00; *p* = 0.01) and CSS (HR 0.98; 95% CI 0.96–1.00; *p* < 0.05). The assessed adipose tissue indices were not significant predictors for OS and CSS. Conclusions: Sarcopenia, a low PMI, and myosteatosis are independent predictors for OS and CSS in patients undergoing radical cystectomy for bladder cancer.

## 1. Introduction

Bladder cancer (BC) is the ninth most common cancer worldwide with a yearly incidence of approximately 430,000 cases [1]. BC is male predominant and is the fourth most common in men in industrial nations such as the USA and Germany [2,3]. For muscle invasive bladder cancer (MIBC) and non-muscle-invasive bladder cancer (NMIBC) refractory to instillation therapy, radical cystectomy (RC) represents the gold standard therapy [4]. Although radical cystectomy is performed with curative intent, patients have a 5-year overall survival (OS) rate of approximately 50–60% [2,5]. Known variables affecting survival after RC include age, histopathologic characteristics, and comorbidity [6,7]. In recent years, sarcopenia was found to be an independent predictor of survival in several tumor entities, including BC [8,9]. Further investigations have shown that aside from sarcopenia, other constitutions of body composition also seem to influence the outcome of various cancer entities [10,11,12].

A wide array of relevant body composition parameters has been identified. Although different nomenclature and thresholds are used throughout the literature, the main parameters include the skeletal muscle index (SMI) [13,14,15,16], the psoas muscle index (PMI) [17], skeletal muscle Hounsfield units (SMHU, also known as skeletal muscle radiation attenuation [SMRA] or skeletal muscle density [SMD]) [14,15,18,19], the subcutaneous fat index (SFI, also known as the subcutaneous adipose tissue index [SATI]) [13,20], the visceral fat index (VFI, also known as the visceral adipose tissue index [VATI]) [16,20], the visceral adipose tissue area (visceral obesity) [13,15,20], and the visceral-to-subcutaneous fat ratio (VSR) [13,16].

Sarcopenia, being the most commonly assessed body composition parameter, was found to be a predictor for survival in several malignancies including ovarian cancer, colorectal cancer, cholangiocarcinoma, gastric cancer, pancreatic cancer, prostate cancer, and BC [13,16,21,22,23,24]. Similarly, the psoas muscle index and myosteatosis are prognostic factors in different tumor entities including urologic tumors [10,17,25,26,27,28,29]. Adipose tissue distribution measurements are more complex to grasp due to their heterogeneity. Increased adipose tissue was associated with both positive and negative effects in previous studies. By observing the adipose tissue distribution and differentiating between short- and long term effects, an “obesity paradox” has been postulated for the protective effect of adipose tissue for long term survival in different tumor entities [30].

In BC research, currently there are only few existing studies on sarcopenia and body composition. Sarcopenia was shown to be a significant predictor for shorter overall and cancer-specific survival [9,31]. The PMI in BC patients has shown diverging significance in past studies [17,32]. Myosteatosis has rarely been investigated in BC patients. One study identified myosteatosis as a risk factor for survival after radical cystectomy, showing a similar effect as described in other tumors [33]. Adipose tissue distribution has not yet found its place in risk stratification for survival in BC patients. Previous studies have indicated a protective effect of adipose tissue on survival outcome. However, no clear significant implications have been made so far [34,35].

The aim of this study was to assess the wide array of commonly established body composition parameters and different thresholds described in the literature as prognostic factors for survival after radical cystectomy due to BC and to place these parameters into context for BC research. Furthermore, we aimed to identify certain body composition prognostic factors for this tumor entity that can be easily assessed and used to evaluate prognosis after RC.

## 2. Patients and Methods

Ethical approval was granted by the institutional ethics committee of the university hospital Regensburg (approval number: 16-101-0095).

### 2.1. Patients

**Figure 1:**
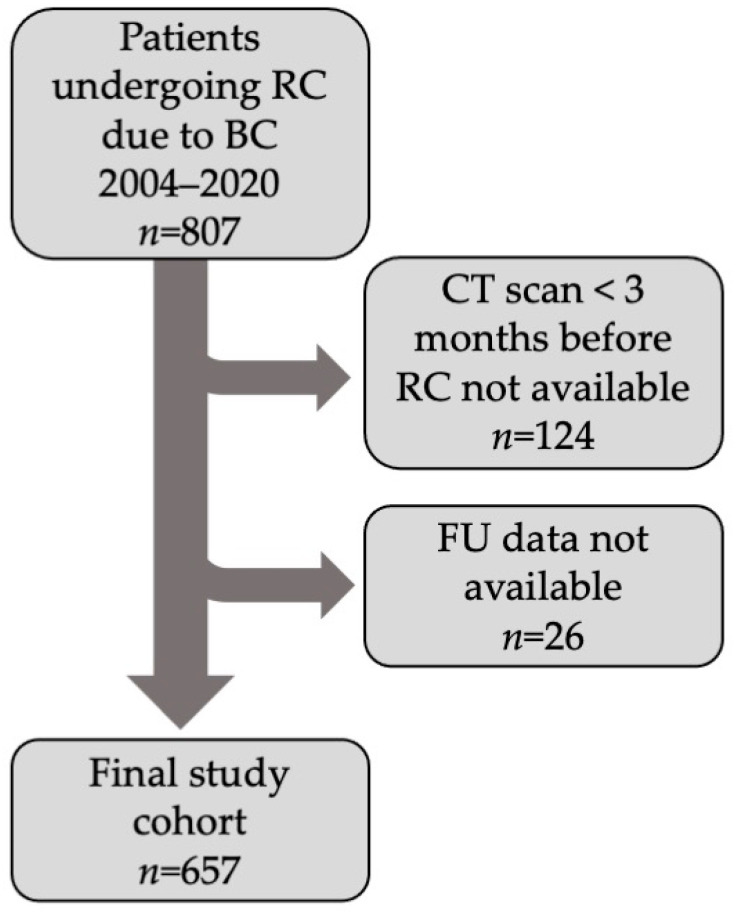
Flowchart showing patient inclusion criteria and final study cohort.

In this single-center retrospective study, 807 patients who underwent RC due to BC between 1 August 2004 and 31 December 2020 were selected. Further inclusion criteria were a preoperative computed tomography (CT), no longer than 3 months prior to RC, and availability of follow-up data. A total of 657 met the eligibility criteria and remained in the study cohort (Figure 1). Patient data, tumor staging information, and comorbidities were collected using in-hospital patient records. Comorbidities were quantified using the Adult Comorbidity Evaluation 27 (ACE-27) scoring model [36]. Tumor staging, age, comorbidities (ACE-27), and perioperative chemotherapy were identified as confounding variables that might influence survival. Follow-up data were collected from hospital patient records, patient telephone interviews, local urologists, and general practitioners. The data collected included last follow-up date, date of death, and cause of death. Overall survival is defined as the time from radical cystectomy to death, regardless of cause of death, and cancer-specific survival was defined as the time from radical cystectomy to death due to bladder cancer progression or metastasis causing death.

### 2.2. Body Composition Measurements

**Figure 2:**
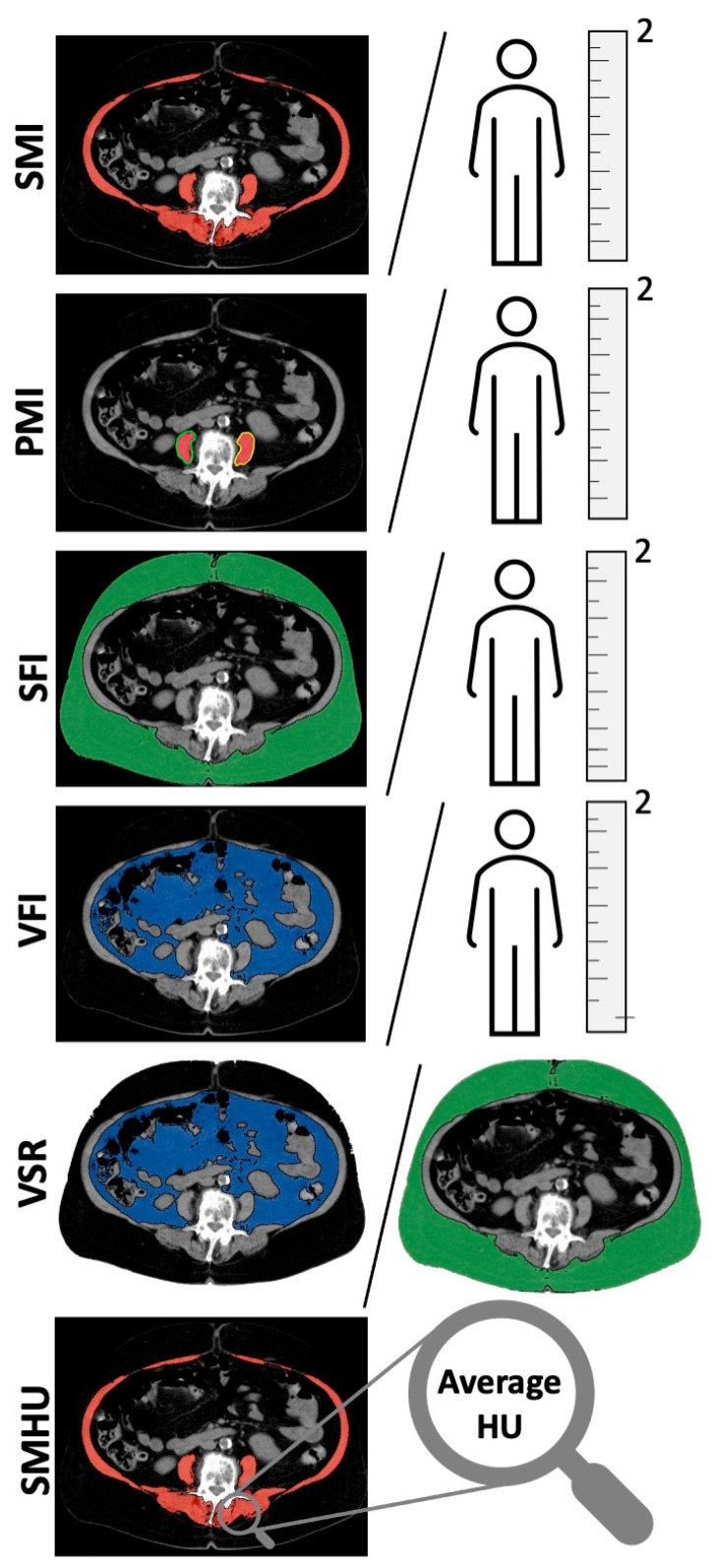
Illustration of different body composition measurements at the height of lumbar vertebra 3. From top to bottom, skeletal muscle index (SMI), psoas muscle index (PMI), subcutaneous fat index (SFI), visceral fat index (VFI), visceral-to-subcutaneous fat ratio (VSR) and skeletal muscle Hounsfield units (SMHU) are shown.

Body composition parameters were measured using preoperative CT scans of the abdomen, at the height of the third lumbar vertebra (L3) using Osirix DICOM viewer software (OsiriX MD version 13.0.0, Pixmeo, Geneva, Switzerland) as previously described [16,17]. All measurements were performed on two consecutive transversal CT images at height of the third lumbar vertebra, on which both transverse processes were visible. The mean measurements were used for further calculations and analyses. Skeletal muscle was identified as Hounsfield units (HUs) of −29 to +150 [37]. Adipose tissue was identified as −150 to −50 HU [38]. Muscles measured at L3 included the psoas, paraspinal, transverse abdominal, external oblique, internal oblique, and rectus abdominis muscles. Intramuscular adipose tissue measurements to determine myosteatosis were performed by determining the mean HU of the skeletal muscle at L3. Different common body composition indices were calculated using the obtained measurements. For the PMI, the average area of one psoas muscle was used as previously described [17]. The SMI and PMI were calculated by normalizing for height in meters squared. The same was performed for the SFI and VFI [16]. The VSR was calculated by dividing visceral fat area by subcutaneous fat area [16]. An overview of measurements and indices used is shown in Figure 2.

The most common values and thresholds that could be found in the literature were used for sarcopenia [14,18,39,40], PMI [17], myosteatosis [14,18,19,24], SFI [11] and visceral obesity [41]. For the VFI and VSR, no suitable validated thresholds were found. For these indices, we used a validated online biomarker optimization software to find optimal cutoffs [42]. The thresholds used for each parameter are shown in Table 1.

### 2.3. Statistical Analysis

Frequencies are presented as absolute numbers and percentages. Continuous data are presented as median with interquartile range (IQR). Differences between groups were analyzed using the Pearson χ^2^ test for dichotomous parameters and the Wilcoxon-Mann–Whitney U test for continuous data. Survival data were analyzed using univariate and multivariate Cox regression. Kaplan–Meier curves were used to illustrate overall (OS) and cancer-specific survival (CSS). Statistical analysis was performed using SPSS software (version 29.0; SPSS Inc., Chicago, IL, USA). Graphs were created using Prism software (Prism 9 for macOS, version 9.4.1).

## 3. Results

### 3.1. Descriptive Data

The distribution of body composition parameters is depicted in Table 1. Patient characteristics for the entire study cohort are depicted in Table 2. The median age was 70 (IQR 63–77) with 167 (25.4%) females and 490 (74.6%) males. In total, 351 (53.4%) patients were alive at the censor date with a median follow-up of 40 months (IQR 15–76 months). As for histopathologic characteristics, 343 (52%) had a pT3 staged tumor or worse, 93 (14.2%) had positive surgical margins, 198 (30.1%) had a positive lymph node status, and 32 (4.9%) had metastases preoperatively. The locations of positive surgical margins were ureteric (*n* = 6, 0.9%), at soft tissue (*n* = 69, 10.5%), at the urethra (*n* = 19, 2.9%) and 2 (0.3%) were undefined in the pathological report.

### 3.2. Relationship of Sarcopenia with Other Parameters

The relationships between sarcopenia, clinical parameters, histopathologic results, and other body composition parameters were assessed and presented in Table 2. Sarcopenia was more common in older patients (*p* < 0.01) and sarcopenic patients had more severe comorbidities according to the ACE-27 scoring model (*p* = 0.01). Higher tumor stages and positive surgical margins were more common in sarcopenic patients (*p* = 0.04 and *p* = 0.02, respectively). There was no significant association between sarcopenia and over- or underweight in the entire cohort (*p* = 0.06). More smokers were not sarcopenic (*p* = 0.05). Sarcopenia (Martin) was significantly associated with other definitions of sarcopenia, myosteatosis (all definitions), and low psoas muscle index (all *p* < 0.01).

### 3.3. Body Composition and Overall Survival

**Figure 3:**
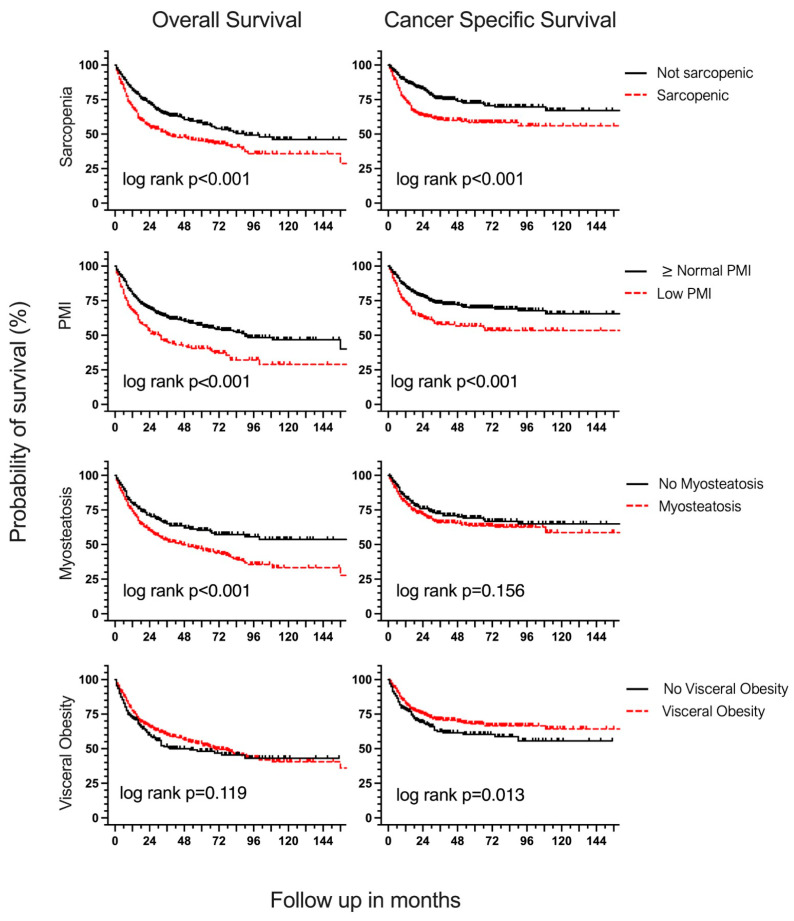
Illustration of Kaplan–Meier overall survival curves of body composition parameters for overall and cancer-specific survival. **Top** to **bottom**: Sarcopenia (Martin), Low PMI, Myosteatosis (Martin), SFI (subcutaneous fat index), VFI (visceral fat index), visceral obesity.

As shown in Table 3, sarcopenia (all definitions), myosteatosis (all definitions), and low PMI were all significant risk factors for overall survival (all *p* < 0.01) analyzed by univariate Cox regression. Sarcopenia defined by Martin et al., low PMI, and myosteatosis defined by Martin et al. were the strongest body composition risk factors for OS identified in this study (Table 3) [14]. The association of these risk factors with OS is illustrated in Figure 3. Sarcopenia (Martin), myosteatosis (Xiao) and SMHU, representing myosteatosis as a continuous variable, and low PMI were significant independent risk factors for OS in a multivariate Cox-regression model adjusted for age, ACE-27, pT-stage, surgical margins, pN-stage, cM-stage, and BMI category (Table 4). None of the analyzed adipose tissue components (SFI, VFI, VSR, and visceral obesity) could be identified as risk factors for OS in our study cohort (Table 3).

### 3.4. Body Composition and Cancer-Specific Survival

All defined thresholds of sarcopenia are significantly associated with CSS in univariate analysis (all *p* < 0.01; Table 3). Myosteatosis as a continuous variable and a low PMI were shown as significant risk factors for CSS in univariate Cox-regression analysis. Visceral obesity (HR 0.70; 95% CI 0.52–0.93; *p* = 0.01) was found to be protective for CSS (Table 3 and Figure 3). In a multivariate Cox-regression analysis adjusted for age, pT-stage, surgical margins, pN-stage, cM-stage, perioperative chemotherapy, and BMI, sarcopenia (Martin) and low PMI and myosteatosis (continuous variable) were found to be independent risk factors for CSS (Table 5). Apart from visceral obesity, which was found to be protective for CSS, none of the other adipose tissue components could be found to be significantly associated with CSS for the entire study cohort.

## 4. Discussion

The aim of our study was to assess different body composition parameters in order to identify those relevant for survival after RC for BC. Our study supports the findings that sarcopenia (Martin) is a strong independent risk predictor for OS (HR 1.30; 95% CI 1.02–1.66; *p* = 0.04) and CSS (HR 1.64; 95% CI 1.19–2.25; *p* < 0.01) as previously described by Mayr et al. and other research groups [9,33]. Low PMI also represents an independent predictor of OS (HR 1.32; 95% CI 1.02–1.70; *p* = 0.03) and CSS (HR 1.41; 95% CI 1.02–1.96; *p* = 0.04) in multivariate analyses. PMI measurements are easier to obtain than the SMI and may even be measured by sonography [44]. Hence, we propose low PMI as a relevant and applicable prognostic factor for patients undergoing RC for BC. Myosteatosis defined by different thresholds was a significant predictor for OS in univariate analysis. Myosteatosis was an independent risk factor for OS as defined by Xiao (HR 1.33; 95% CI 1.00–1.76; *p* < 0.05). Myosteatosis as a continuous variable was shown to be a risk factor for OS (HR 0.98; 95% CI 0.97–1.00, *p* = 0.01) and CSS (HR 0.98; 95% CI 0.96–1.00; *p* < 0.05). None of the indices involving adipose tissue measurements that were examined in this study could be identified as independent prognostic factors.

### 4.1. Choice of Body Composition Thresholds

We aimed to assess commonly used body composition parameters in cancer research. We identified those often referred to in the literature and where possible, those assessed in sizable cohorts (Table 1) [14,18,19,39,43]. Where possible, thresholds that were assessed in cohorts with urothelial cancer were utilized [17]. By using different thresholds for analyses of body composition, statistical analysis yielded different results (Table 3, Table 4 and Table 5). This underlines the heterogeneity of thresholds used in research due to differing study populations, cancer entities, and baseline characteristics. We found the Martin criteria for sarcopenia, the Kasaharas threshold for low PMI, and the Xiaos threshold for myosteatosis to be most significant for our study cohort (Table 3, Table 4 and Table 5).

### 4.2. Sarcopenia

Different definitions of sarcopenia were used in data analysis (Table 1). While statistical findings between them remain similar, the Martin criteria are most commonly used in the existing literature and are adjusted for gender and BMI [14]. In relevant earlier studies on patients undergoing RC for BC, sarcopenia was also defined by the Martin criteria [9,45]. Nonetheless, the Martin criteria recently have been discussed controversially because the thresholds for men are discontinuous, dependent on BMI [46]. Nevertheless, no consensus was found to this date. Using the Martin criteria, 52% of patients of the entire cohort were defined as sarcopenic. This is in line with a Japanese single-center study published in 2016, where 48% of patients were sarcopenic [45]. In another Japanese single-center study by, 39% of patients were classified as sarcopenic [33]. A limitation to comparability is the use of another threshold for sarcopenia not considering BMI. A key finding of our current study is the independent association of sarcopenia and OS and CSS (Table 4 and Table 5 and Figure 3). These results align with the findings of Mayr et al. in 2018 where sarcopenia was also an independent predictor for CSS (HR 1.42; 95% CI 1.00–2.02; *p* < 0.05) and OS (HR 1.43; 95% CI 1.09–1.87; *p* = 0.01) [9]. Our current results also align with findings made in earlier studies by Psutka et al., Hirasawa et al., and Yamashita et al. [31,33,45]. Of note, the study cohorts of Psutka and Hirasawa were both limited by sample size. The study cohort by Mayr et al. had a sufficient sample size with 500 patients and 234 events in total [9]. However, it was a multicenter, multinational study. The current study underlines the suggested findings in a large single-center cohort of 657 patients.

### 4.3. Psoas Muscle Index

We have shown that a low PMI is an independent risk factor for OS and CSS (Table 4 and Table 5). Only few studies on the PMI exist in patients undergoing RC due to BC. The PMI is often used as a shortcut-index to determine sarcopenia [32]. Although we have shown that sarcopenia is significantly associated with a low PMI (Table 2), these terms should not be used in a synonymic manner. Not all patients with a low PMI are identified as sarcopenic (Martin) and vice versa (Table 2). Nevertheless, we do believe that the PMI is a very useful prognostic measurement that can also be easily obtained via sonography during patient consultation [44].

In a single-center retrospective study with a sizable study cohort of 441 patients, Stangl-Kremser et al. did not find a low PMI to be a statistically significant independent risk factor for OS, although a statistical trend was shown (HR 1.59; 95% CI 0.98–2.59; *p* = 0.06) [35]. A major difference between the current study is the thresholds used for the PMI. Stangl-Kremser et al. used thresholds determined in a healthy population with a mean age of 31 years [47]. In the current study, we used threshold values described by Kasahara et al. in a population with advanced bladder cancer with a mean age of 61.9 years [17]. The latter is better comparable to patients undergoing RC due to bladder cancer. Kasahara et al. identified a low PMI as a risk factor for shorter survival (log-rank *p* = 0.015). While a low PMI is a strong independent predictor of OS and CSS after radical cystectomy (Figure 3), it is also one of the simplest measurements to take. Thus, we propose the PMI as a diagnostic and prognostic tool that should be used on a day-to-day basis during patient consultation.

### 4.4. Myosteatosis

To date, Myosteatosis has barely been mentioned in the literature as a prognostic factor in patients with BC. In other tumor entities, myosteatosis has found its place as a risk factor. For instance, Martin et al. have identified myosteatosis as an independent risk for OS in a large cohort of 1473 patients with lung and gastrointestinal cancer [14]. For BC, only one comparable study was found. Yamashita et al. assessed 123 patients who underwent RC for BC [33]. They found that myosteatosis was a strong independent predictor for CSS (HR 3.53; 95% CI 1.30–12.50; *p* = 0.04) and not for OS (*p* = 0.10). In our study, myosteatosis had a statistically significant association in univariate analyses with OS in all definitions used, whereby the definition posed by Martin et al. was the strongest risk factor (HR 1.63; 95% CI 1.27–2.10; *p* < 0.01; Figure 2) [14]. An independent association in multivariate analysis could be shown for OS if using myosteatosis (SMHU) as a continuous variable and the definition by Xiao et al. (Table 4) [18]. Regarding the continuous variable, one must note that lower SMHU values represent a higher adipose tissue content of muscle, thus myosteatosis. An independent risk by myosteatosis for CSS could be shown using the continuous variable (HR 0.98; 95% CI 0.96–1.0; *p* < 0.05, Table 5). The thresholds for myosteatosis in our current study were different to those used by Yamashita et al. (SMD < 38.5 HU for men and SMD < 28.6 HU for women) [33]. The fact that the continuous variable is an independent statistically significant risk factor for OS and CSS may suggest that we did not find an appropriate threshold for our dataset by using previously mentioned thresholds.

### 4.5. Adipose Tissue Indices

To the best of our knowledge, no comparable study assessing adipose tissue distribution indices in patients undergoing RC exists to this date. We examined adipose tissue parameters that have been used in body composition research for other diseases [11,15,16,41]. In our study, we did not find statistically significant associations between adipose tissue indices (SFI, VFI, VSR) and OS or CSS in the entire cohort (Table 3). Visceral obesity seemed to have a protective effect for CSS (HR 0.70; 95% CI 0.52–0.93; *p* = 0.01) in univariate analysis. Psutka et al. made implications of adipose tissue having protective effects in a study published in 2015 of 262 patients undergoing RC. Here, the total fat area at the height of L3 was measured and the whole-body fat mass was calculated. Psutka et al. conclude “…among patients with normal muscularity there is a trend toward improved survival in those with increasing weight and adiposity…” [34]. The protective value of adipose tissue was also identified by Martini et al. in a study of 70 patients with advanced urothelial cancer treated with immune checkpoint inhibitors [10]. High VFI was significantly associated with improved progression-free survival (HR 1.76, *p* = 0.04) and showed a trend toward longer OS. High SFI was significantly associated with prolonged OS (HR 1.99, *p* = 0.043). In another study conducted by Stangl-Kremser et al. on 68 patients treated with radiation for BC, no association with survival could be found for visceral fat area, subcutaneous fat area, and visceral-to-subcutaneous fat ratio, i.e., similar to our findings [35].

### 4.6. Limitations

Our study has several limitations. Due to the retrospective nature of our study, we collected data from 657 patients in a non-consecutive manner. The follow-up information we were able to retrieve was heterogenic depending on reachability of either patients, general practitioners, or local urologists. Therefore, we were not able to reliably collect information such as progression-free survival. Furthermore, due to the retrospective data, our study lacks other frailty measurements such as hand-grip strength, performance status on questionnaires, or other potentially interesting examinations such as blood analysis for inflammation or nutritional status (albumin levels). Although having selected patients undergoing RC due to BC, there is still a certain heterogeneity in our cohort. Tumor stages ranging from pTa up to pT4 were all included; also, patients who received neoadjuvant chemotherapy were included. However, neoadjuvant chemotherapy was not associated with sarcopenia in our cohort (*p* = 0.084, Table 2). Due to the long retrospective period of our study, we have a low rate of neoadjuvant therapies (7.2%) compared to recent cystectomy studies. In the early years of our study cohort, barely any neoadjuvant chemotherapy was given, whereas in the later years rates exceeded 20%. Our study cohort has a high rate of positive surgical margins (14.2%) compared to the literature, where the rates are reported at 5% [48]. This may be due to the high portion of pT3 (35%) and pT4 (17%) in our study. In the literature, much lower rates of pT3 (22%) and pT4 (7%) are described [49]. There is a need for a prospective study with follow-up appointments, including CT scans for further body composition analysis during the follow-up period.

## 5. Conclusions

To the best of our knowledge, this study represents the largest cohort assessed for body composition parameters in patients undergoing RC due to BC.

Sarcopenia and the psoas muscle index proved to be strong independent predictors for OS and CSS. The psoas muscle index can be used as a simple prognostic tool during patient consultation. Myosteatosis is an independent predictor for OS in this patient cohort. Due to the risks posed by sarcopenia, we are planning a preoperative exercise and nutritional support program in collaboration with the department of oncology in our center, similar to the program proposed by Yamamoto et al. [50]. Body composition parameters such as myosteatosis, subcutaneous and visceral fat indices, visceral-to-subcutaneous fat ratio, and visceral obesity have only been investigated in a low number of BC patients up to this date. Hence, there is a lack of comparable studies, making it difficult to place these findings into context. There is a need for further large studies assessing the effect of adipose tissue distribution in BC. Outside of BC research, sarcopenia and body composition research are a rapidly growing field. As demonstrated in our study, there are several definitions and thresholds for different measurements in the literature. There is an urgent need for unification and consensus on thresholds to increase comparability between studies and tumor entities.

## Figures and Tables

**Table 1:** Computed tomography-derived body composition measures and thresholds used and calculated for the entire cohort of 657 bladder cancer patients who underwent radical cystectomy.

Body Composition Measurement	Frequency *n* (%)
**BMI**	
Normal (18.5–24.9 kg/m^2^)	204 (31.1)
Severely Underweight (<16 kg/m^2^)	2 (0.3)
Underweight (17–18.4 kg/m^2^)	14 (2.1)
Overweight (25–29.9 kg/m^2^)	279 (42.5)
Obese I (30–34.9 kg/m^2^)	120 (18.3)
Obese II (35–39.9 kg/m^2^)	27 (4.1)
Adipositas per Magna (≥40 kg/m^2^)	11 (1.7)
**Sarcopenia**	
**SMI (Martin)** [14]	340 (51.8)
Males: SMI < 43 cm^2^/m^2^ if BMI < 25 kg/m^2^ or SMI < 53 cm^2^/m^2^ if BMI ≥ 25 kg/m^2^, females: SMI < 41 cm^2^/m^2^	
**SMI (Caan)** [43]	391 (59.5)
Males: SMI < 52.3 cm^2^/m^2^ if BMI < 30 kg/m^2^ or SMI < 54.3 cm^2^/m^2^ if BMI ≥ 30 kg/m^2^, females: SMI < 38.6 cm^2^/m^2^ if BMI < 30 kg/m^2^ or SMI < 46.6 cm^2^/m^2^ if BMI ≥ 30 kg/m^2^	
**SMI (Prado)** [39]	368 (56)
Males: SMI ≤ 52.4 cm^2^/m^2^, females: SMI ≤ 38.5 cm^2^/m^2^	
**SMI (Lancet Oncology Consensus)** [40]	434 (66.1)
Males: SMI < 55 cm^2^/m^2^, females: SMI < 39 cm^2^/m^2^	
**Psoas Muscle Index**	
**Low PMI (Kasahara)** [17]	224 (34.1)
Males: <2.49 cm^2^/m^2^, females: <2.07 cm^2^/m^2^	
**Myosteatosis**	
**SMHU (Derstine)** [19]	474 (72.1)
Males: <38.5 HU, Females: <34.3 HU	
**SMHU (Martin, Lurje)** [14,24]	430 (65.4)
Patients with BMI < 25 kg/m^2^: <41 HU, Patients with BMI ≥ 25 kg/m^2^: <33 HU	
**SMHU (Xiao)** [18]	414 (63)
Males: <35.5 HU, Females: <32.5 HU	
**High SFI** [11]	300 (45.7)
Males: ≥50.0 cm^2^/m^2^. females: ≥42.0 cm^2^/m^2^	
**Visceral Obesity** [41]	436 (66.4)
Males: VFA > 160 cm^2^, females: >80 cm^2^	
**High VFI (Engelmann)**	342 (52.1)
Males: >74.34 cm^2^/m^2^, females: >28.38 cm^2^/m^2^	
**High VSR (Engelmann)**	308 (46.9)
Males: >1.421, females >0.4255	

SFI: subcutaneous fat index, VFI: visceral fat index, VSR: visceral-to-subcutaneous fat ratio, SMI: skeletal muscle index, PMI: psoas muscle index, SMHU: skeletal muscle Hounsfield units, BMI: body mass index.

**Table 2:** Clinicopathologic parameters in the entire radical cystectomy cohort and relationship between sarcopenia (Martin) and clinicopathologic and body composition parameters.

Characteristic	Entire Cohort *n* = 657 (%)	Not Sarcopenic *n* = 317 (48.2%)	Sarcopenic *n* = 340 (52.8%)	*p*-Value
Age Median (IQR)	70 (63–77)	67 (60–74)	73 (66–79)	**<0.01**
Gender (male)	490 (74.6)	245 (77.3)	245 (72.1)	0.12
Smoker (former or current)	349 (53.1)	180 (56.8)	168 (49.4)	0.06
ACE-27				
None	111 (16.9)	69 (21.8)	42 (12.4)	**0.01**
Mild	209 (31.8)	92 (29)	117 (34.4)	
Moderate	205 (31.2)	93 (29.3)	112 (32.9)	
Severe	132 (20.1)	63 (19.9)	69 (20.3)	
T-Stage				
pTa, pT1, pTis	92 (14)	50 (15.8)	42 (12.4)	**0.04**
pT2	222 (33.8)	120 (37.9)	102 (30)	
pT3	229 (34.9)	99 (31.2)	130 (38.2)	
pT4	114 (17.4)	48 (15.1)	66 (19.4)	
Positive R-Stage	93 (14.2)	34 (10.8)	59 (17.4)	**0.02**
N-Stage				
N0	439 (66.8)	216 (68.1)	223 (65.6)	0.66
N+	198 (30.1)	93 (29.3)	105 (30.9)	
Nx	20 (3)	8 (2.5)	12 (3.5)	
Preoperative cM-Stage				
cM+	32 (4.9)	14 (4.4)	18 (5.3)	0.60
Perioperative Chemotherapy				
Neoadjuvant	47 (7.2)	17 (5.4)	30 (8.9)	0.08
Adjuvant	94 (14.3)	51 (16.5)	43 (13)	0.21
BMI Category				
Normal	204 (31.1)	100 (31.5)	104 (30.6)	0.06
Underweight	16 (2.4)	3 (0.9)	13 (3.8)	
Overweight	437 (66.7)	214 (67.5)	223 (65.6)	
Sarcopenia				
(Caan) [43]	391 (59.5)	80 (25.2)	311 (91.5)	**<0.01**
(Prado) [39]	368 (56)	64 (20.2)	304 (89.4)	**<0.01**
(L.O. Consensus) [40]	434 (66.1)	107 (33.8)	327 (96.2)	**<0.01**
Myosteatosis				
(Derstine) [19]	473 (72)	206 (65)	267 (79)	**<0.01**
(Martin, Lurje) [14,24]	429 (65.3)	177 (55.8)	252 (74.6)	**<0.01**
(Xiao) [18]	413 (62.9)	171 (53.9)	242 (71.6)	**<0.01**
Low PMI	224 (34.1)	50 (15.8)	174 (51.2)	**<0.01**
High SFI	300 (45.7)	153 (48.3)	147 (43.2)	0.20
Visceral Obesity	436 (66.4)	211 (66.6)	225 (66.2)	0.92
High VFI	342 (52.1)	168 (53)	174 (51.2)	0.64
High VSR	308 (46.9)	142 (44.8)	166 (48.8)	0.30

ACE: adult comorbidity evaluation, T-stage: tumor stage, R-stage: surgical margin, N-stage: nodal status, cM-Stage: preoperative known metastasis, SFI: subcutaneous fat index, VFI: visceral fat index, VSR: visceral-to-subcutaneous fat ratio, PMI: psoas muscle index, BMI: body mass index.

**Table 3:** Univariate Cox-regression analysis for overall survival and cancer-specific survival in the 657 radical cystectomy patients.

	OS			CSS		
Characteristic	HR	95% CI	*p*	HR	95% CI	*p*
Age (Years, continuous)	**1.05**	**1.03–1.06**	**<0.01**	**1.02**	**1.01–1.04**	**<0.01**
Gender (ref. Male)	1.24	0.96–1.60	0.10	**1.47**	**1.08–2.01**	**0.02**
Smoker (ref. no)	**0.79**	**0.63–0.98**	**0.04**	0.75	0.57–1.00	0.05
ACE-27 (Ref. ACE-27 0)						
Mild	1.22	0.84–1.77	0.29	1.03	0.67–1.57	0.90
Moderate	**1.61**	**1.13–2.30**	**<0.01**	1.07	0.70–1.63	0.75
Severe	**2.17**	**1.50–3.15**	**<0.01**	1.40	0.89–2.18	0.14
T-Stage (Ref. pTa, pT1, pTis)						
pT2	0.92	0.60–1.43	0.72	1.34	0.63–2.84	0.45
pT3	**3.01**	**2.02–4.48**	**<0.01**	**6.40**	**3.22–12.71**	**<0.01**
pT4	**4.20**	**2.75–6.42**	**<0.01**	**11.03**	**5.48–22.23**	**<0.01**
Positive R-Stage (Ref. R0)	**2.68**	**2.02–3.56**	**<0.01**	**3.52**	**2.53–4.90**	**<0.01**
N-Stage (Ref. N0)						
N+	**2.72**	**2.15–3.44**	**<0.01**	**4.58**	**3.40–6.19**	**<0.01**
Nx	**3.26**	**1.88–5.64**	**<0.01**	**5.25**	**2.78–9.91**	**<0.01**
Positive cM-stage (Ref. cM0)	**4.14**	**2.70–6.34**	**<0.01**	**6.86**	**4.42–10.64**	**<0.01**
Perioperative Chemotherapy (Ref.: No)	1.21	0.92–1.58	0.17	**1.66**	**1.22–2.27**	**<0.01**
BMI Category (Ref. Normal)						
Severely Underweight	**7.17**	**1.75–29.35**	**0.01**	5.36	0.74–39.01	0.10
Underweight	1.72	0.87–3.41	0.12	1.83	0.84–3.97	0.13
Overweight	0.80	0.62–1.04	0.10	**0.71**	**0.51–0.98**	**0.04**
Obese I	0.77	0.55–1.07	0.12	**0.63**	**0.41–0.96**	**0.03**
Obese II	1.04	0.59–1.81	0.90	**0.31**	**0.10–0.97**	**0.04**
Adipositas per Magna	0.62	0.23–1.69	0.35	0.45	0.11–1.82	0.26
Sarcopenia (Ref. absence)						
Sarcopenia (Martin) [14]	**1.59**	**1.27–2.00**	**<0.01**	**1.87**	**1.40–2.51**	**<0.01**
Sarcopenia (Caan) [43]	**1.36**	**1.08–1.72**	**<0.01**	**1.53**	**1.14–2.07**	**<0.01**
Sarcopenia (Prado) [39]	**1.40**	**1.12–1.76**	**<0.01**	**1.57**	**1.17–2.10**	**<0.01**
Sarcopenia (L.O. Consensus) [40]	**1.43**	**1.12–1.83**	**<0.01**	**1.64**	**1.19–2.26**	**<0.01**
Myosteatosis (Ref. absence)						
(Derstine) [19]	**1.54**	**1.18–2.01**	**<0.01**	1.14	0.83–1.57	0.41
(Martin, Lurje) [14,24]	**1.63**	**1.27–2.10**	**<0.01**	1.26	0.93–1.71	0.14
(Xiao) [18]	**1.54**	**1.21–1.96**	**<0.01**	1.10	0.82–1.48	0.51
Myosteatosis (HU, continuous)	**0.97**	**0.96–0.98**	**<0.01**	**0.98**	**0.97–1.00**	**0.04**
Low PMI (Ref. High PMI)	**1.67**	**1.33–2.10**	**<0.01**	**1.85**	**1.39–2.46**	**<0.01**
High SFI (Ref. Low SFI)	0.97	0.77–1.209	0.76	0.82	0.62–1.10	0.19
Visceral Obesity (Ref. No)	0.83	0.66–1.05	0.12	**0.70**	**0.52–0.93**	**0.01**
High VFI (Ref. low VFI)	0.96	0.77–1.20	0.72	0.77	0.58–1.02	0.07
High VSR (Ref. low VSR)	1.16	0.93–1.45	0.20	0.97	0.73–1.29	0.84

Ref.: reference, CI: confidence interval, ACE: adult comorbidity evaluation, T-stage: tumor stage, R-stage: surgical margin, N-stage: nodal status, cM-Stage: preoperative known metastasis, SFI: subcutaneous fat index, VFI: visceral fat index, VSR: visceral-to-subcutaneous fat ratio, PMI: psoas muscle index, BMI: body mass index.

**Table 4:** Multivariate Cox-regression analyses for different body composition measurements for OS in the 657 patients who underwent radical cystectomy.

	MV Model with Sarcopenia (Martin)			MV Model with Low PMI			MV Model with Myosteatosis (Xiao)			MV Model with Myosteatosis (Cont.)		
Characteristic	HR	95% CI	*p*	HR	95% CI	*p*	HR	95% CI	*p*	HR	95% CI	*p*
Age (Years, continuous)	**1.03**	**1.02–1.05**	**<0.01**	**1.03**	**1.02–1.05**	**<0.01**	**1.03**	**1.02–1.05**	**<0.01**	**1.03**	**1.01–1.04**	**<0.01**
ACE-27 (Ref. ACE-27 0)												
Mild	0.86	0.59–1.25	0.43	0.87	0.59–1.27	0.47	0.84	0.57–1.23	0.37	0.85	0.58–1.24	0.40
Moderate	1.24	0.86–1.80	0.25	1.26	0.87–1.83	0.23	1.18	0.81–1.73	0.38	1.19	0.82–1.73	0.37
Severe	**1.57**	**1.06–2.32**	**0.02**	**1.54**	**1.05–2.29**	**0.03**	**1.49**	**1.00–2.22**	**<0.05**	**1.50**	**1.01–2.23**	**<0.05**
T-Stage (Ref. pTa, pT1, pTis)												
pT2	0.80	0.52–1.25	0.33	0.79	0.51–1.22	0.29	0.78	0.50–1.22	0.28	0.78	0.50–1.21	0.26
pT3	**2.06**	**1.35–3.13**	**<0.01**	**2.10**	**1.38–3.19**	**<0.01**	**2.02**	**1.32–3.07**	**0.00**	**1.99**	**1.31–3.04**	**<0.01**
pT4	**2.23**	**1.39–3.58**	**<0.01**	**2.23**	**1.39–3.59**	**<0.01**	**2.20**	**1.37–3.53**	**0.00**	**2.19**	**1.36–3.53**	**<0.01**
Positive R-Stage (Ref. R0)	**1.40**	**1.01–1.94**	**<0.05**	**1.41**	**1.02–1.96**	**0.04**	**1.39**	**1.00–1.93**	**0.05**	1.38	0.99–1.92	0.06
N-Stage (Ref. N0)												
N+	**1.77**	**1.34–2.33**	**<0.01**	**1.72**	**1.30–2.27**	**<0.01**	**1.82**	**1.37–2.40**	**<0.01**	**1.77**	**1.34–2.33**	**<0.01**
Nx	1.43	0.80–2.55	0.23	1.44	0.81–2.58	0.22	1.39	0.78–2.49	0.27	1.39	0.78–2.47	0.27
Positive cM-stage (Ref. cM0)	**2.58**	**1.62–4.10**	**<0.01**	**2.52**	**1.58–4.01**	**<0.01**	**2.47**	**1.55–3.93**	**<0.01**	**2.56**	**1.61–4.07**	**<0.01**
BMI Category (Ref. Normal)												
Severely Underweight	**6.67**	**1.56–28.46**	**0.01**	**6.54**	**1.53–27.92**	**0.01**	**8.63**	**2.03–36.91**	**<0.01**	**8.98**	**2.09–38.50**	**<0.01**
Underweight	1.56	0.78–3.15	0.21	1.42	0.70–2.89	0.33	1.74	0.85–3.53	0.13	1.64	0.81–3.31	0.18
Overweight	**0.72**	**0.55–0.95**	**0.02**	0.79	0.60–1.04	0.09	**0.72**	**0.55–0.94**	**0.02**	**0.71**	**0.55–0.93**	**0.01**
Obese I	0.88	0.63–1.25	0.48	0.93	0.65–1.32	0.68	0.78	0.55–1.10	0.16	0.76	0.53–1.08	0.12
Obese II	1.57	0.89–2.78	0.12	1.67	0.93–2.97	0.08	1.37	0.77–2.44	0.28	1.24	0.69–2.23	0.48
Adipositas per Magna	0.55	0.20–1.53	0.25	0.58	0.21–1.63	0.30	0.45	0.16–1.26	0.13	0.39	0.14–1.11	0.08
Sarcopenia (Martin) [14] (Ref. absence)	**1.30**	**1.02–1.66**	**0.04**	-	-	-	-	-	-	-	-	-
Low PMI (Ref. High PMI)	-	-	-	**1.32**	**1.02–1.70**	**0.03**	-	-	-	-	-	-
Myosteatosis (Xiao) [18] (Ref. absence)	-	-	-	-	-	-	**1.32**	**1.00–1.75**	**<0.05**	-	-	-
Myosteatosis (HU, continuous)	-	-	-	-	-	-	-	-	-	**0.98**	**0.97–1.00**	**0.01**

Multivariate analyses adjusted for Age, ACE-27, T-Stage, R-Stage, N-Stage, cM-Stage, BMI Category. Ref.: reference, CI: confidence interval, ACE: adult comorbidity evaluation, T-stage: tumor stage, R-stage: surgical margin, N-stage: nodal status, cM-Stage: preoperative known metastasis, SFI: subcutaneous fat index, VFI: visceral fat index, VSR: visceral-to-subcutaneous fat ratio, PMI: psoas muscle index, BMI: body mass index.

**Table 5:** Multivariate Cox-regression analyses for different body composition measurements for CSS in the 657 patients who underwent radical cystectomy.

Characteristic	MV Model with Sarcopenia (Martin)			MV Model with Low PMI			MV Model with Myosteatosis (Cont.)		
	HR	95% CI	*p*	HR	95% CI	*p*	HR	95% CI	*p*
Age (Years, continuous)	1.01	0.99–1.03	0.30	1.01	0.99–1.03	0.21	1.01	0.99–1.03	0.43
T-Stage (Ref. pTa, pT1, pTis)									
pT2	1.23	0.58–2.63	0.59	1.20	0.56–2.55	0.64	1.16	0.54–2.47	0.71
pT3	**4.27**	**2.09–8.73**	**<0.01**	**4.39**	**2.14–8.99**	**<0.01**	**4.11**	**2.01–8.44**	**<0.01**
pT4	**5.21**	**2.42–11.18**	**<0.01**	**5.17**	**2.41–11.10**	**<0.01**	**5.03**	**2.34–10.81**	**<0.01**
Positive R-Stage (Ref. R0)	**1.61**	**1.09–2.39**	**0.02**	**1.60**	**1.08–2.38**	**0.02**	**1.57**	**1.06–2.33**	**0.03**
N-Stage (Ref. N0)									
N+	**2.43**	**1.68–3.51**	**<0.01**	**2.34**	**1.62–3.40**	**<0.01**	**2.40**	**1.66–3.48**	**<0.01**
Nx	**2.15**	**1.10–4.20**	**0.03**	**2.29**	**1.17–4.50**	**0.02**	**2.14**	**1.09–4.18**	**0.03**
Positive cM-stage (Ref. cM0)	**2.63**	**1.59–4.35**	**<0.01**	**2.56**	**1.54–4.24**	**<0.01**	**2.62**	**1.58–4.34**	**<0.01**
Perioperative Chemotherapy (Ref. no)	0.77	0.53–1.11	0.16	0.77	0.53–1.10	0.15	0.80	0.55–1.15	0.23
BMI Category (Ref. Normal)									
Severely Underweight	2.20	0.29–17.04	0.45	2.36	0.30–18.23	0.41	3.28	0.42–25.39	0.25
Underweight	1.56	0.65–3.77	0.32	1.25	0.51–3.05	0.63	1.47	0.60–3.58	0.40
Overweight	**0.62**	**0.44–0.87**	**0.01**	**0.70**	**0.49–0.98**	**0.04**	**0.62**	**0.44–0.87**	**0.01**
Obese I	0.86	0.55–1.34	0.51	0.88	0.56–1.39	0.58	0.69	0.44–1.08	0.11
Obese II	0.58	0.18–1.85	0.35	0.63	0.20–2.05	0.45	0.45	0.14–1.46	0.18
Adipositas per Magna	0.60	0.15–2.49	0.49	0.61	0.15–2.55	0.50	0.38	0.09–1.62	0.19
Sarcopenia (Martin) [14] (Ref. absence)	**1.64**	**1.19–2.25**	**<0.01**	-	-	-	-	-	-
Low PMI (Ref. High PMI)	-	-	-	**1.41**	**1.02–1.96**	**0.04**	-	-	-
Myosteatosis (HU, continuous)	-	-	-	-	-	-	**0.98**	**0.96–1.00**	**<0.05**

Multivariate analyses adjusted for Age, T-Stage, R-Stage, N-Stage, cM-Stage, perioperative chemotherapy, BMI Category. Ref.: reference, CI: confidence interval, ACE: adult comorbidity evaluation, T-stage: tumor stage, R-stage: surgical margin, N-stage: nodal status, cM-Stage: preoperative known metastasis, SFI: subcutaneous fat index, VFI: visceral fat index, VSR: visceral-to-subcutaneous fat ratio, PMI: psoas muscle index, BMI: body mass index.

## Data Availability

The data can be shared upon request.
